# Enzymatic Kinetic Resolution of *tert*-Butyl 2-(1-Hydroxyethyl)phenylcarbamate, A Key Intermediate to Chiral Organoselenanes and Organotelluranes

**DOI:** 10.3390/molecules16098098

**Published:** 2011-09-20

**Authors:** Leandro Piovan, Monica D. Pasquini, Leandro H. Andrade

**Affiliations:** Institute of Chemistry, University of São Paulo, Av. Prof. Lineu Prestes 748, SP 05508-900, São Paulo, Brazil

**Keywords:** alcohols, carbamates, lipases, kinetic resolution, enatiopure

## Abstract

The enzymatic kinetic resolution of *tert*-butyl 2-(1-hydroxyethyl)phenylcarbamate *via* lipase-catalyzed transesterification reaction was studied. We investigated several reaction conditions and the carbamate was resolved by *Candida antarctica* lipase B (CAL-B), leading to the optically pure (*R*)- and (*S*)-enantiomers. The enzymatic process showed excellent enantioselectivity (*E* > 200). (*R*)- and (*S*)-*tert*-butyl 2-(1-hydroxyethyl)phenylcarbamate were easily transformed into the corresponding (*R*)- and (*S*)-1-(2-aminophenyl)ethanols.

## 1. Introduction

**Scheme 1:**
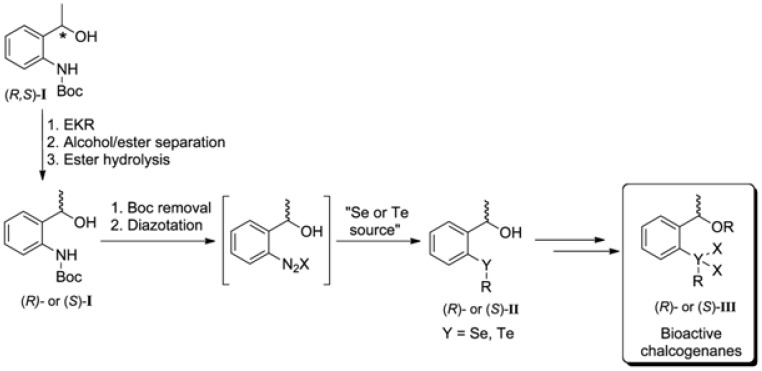
Synthetic route to bioactive chalcogenanes [5,6,9,12].

Selenium- and tellurium-containing compounds have drawn the attention of the scientific community due to their biological properties [1,2,3,4]. Notwithstanding the intense activity in the field of selenium and tellurium chemistry over the last three decades, organometallic reagents are commonly employed on the preparation of organo-selenium and -tellurium compounds. Moreover, hypervalent organoselenium(IV) compounds (organoselenanes) and organotellurium(IV) compounds (organotelluranes) have been investigated as cysteine protease [5,6,7,8], protein tyrosine phosphatase [9] and poliovirus 3C proteinase inhibitors [10]. Considering the biological activities of organoselenanes and telluranes, we have described chemoenzymatic methodologies to synthesize selenium compounds without employing organolithium or organomagnesium reagents [11,12]. Herein, we report the preparation of enantiopure organochalcogenane precursors, (*R*)- and (*S*)-*tert*-butyl 2-(1-hydroxyethyl)phenylcarbamate, employing enzymatic kinetic resolution (EKR) catalyzed by lipases. The chiral building blocks [(*R*)-**I** and (*S*)-**I**] could be applied as advanced synthetic intermediates of organotelluranes and organoselenanes **III**, containing an asymmetric center (Scheme 1). It is possible to transform (*R*)-**I** and (*S*)-**I** into their respective arene diazonium salts, followed by a reaction with a nucleophilic selenium/tellurium specie to give selenides/tellurides **II**, direct precursors of selenanes and telullaranes [12].

## 2. Results and Discussion

**Scheme 2:**
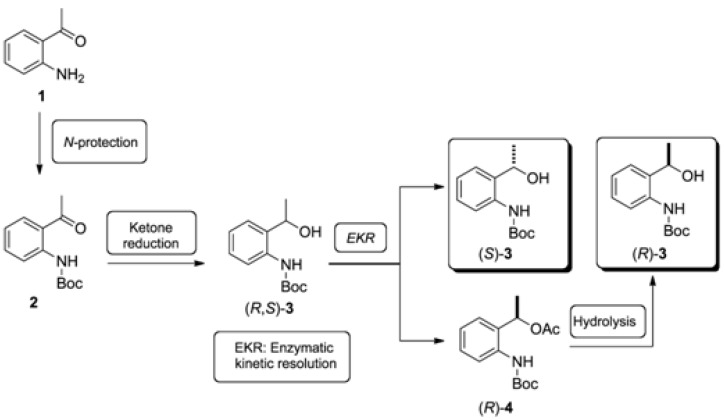
Synthetic route to enantiopure *tert*-butyl 2-(1-hydroxyethyl)phenylcarbamates.

As outlined in Scheme 2, chiral building blocks (*R*)-**3** and (*S*)-**3** could be synthesized from commercially available 1-(2-aminophenyl)ethanone (**1**). Initially, the amine protection leads to the *N*-Boc-protected arylketone **2**, which by reduction of the ketone group affords (*R*,*S*)-**3**. Then, the latter could be submitted to an enzymatic kinetic resolution (EKR) and, at the end of the process, both enantiomers could be easily separated.

### 2.1. Synthesis of the (R,S)-tert-butyl 2-(1-Hydroxyethyl)phenylcarbamate *(**3**)*

Several methods were evaluated to synthesize *tert*-butyl (2-acetylphenyl)carbamate (**2**) (Table 1). The protection of amine group was carried out by reacting 1-(2-aminophenyl)ethanone (**1**) with *tert*-butyl dicarbonate [(Boc)_2_O]. For example, by using dichloromethane (CH_2_Cl_2_) as solvent and DMAP as additive, after 24 h at room temperature, compound **2** was obtained in 60% yield (Entry 1). It is worth mentioning that the intermediate **1a** was observed, then easily transformed to the compound **2** [13]. A second method, employing THF as solvent and under reflux was evaluated. However, a slight yield improvement (compound **2**, 67%) was observed with a shorter reaction time, 12 h (Entry 2). Other reaction conditions were evaluated; however, the yields were lower than 40% (Entries 3–5). Next, we decided to apply the second method (Entry 2) to prepare the compound **2** in a preparative scale (5 mmol).

**Table 1:** Synthesis of *tert*-butyl (2-acetylphenyl)carbamate (**2**). 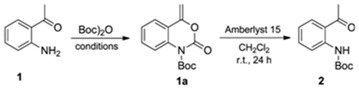

Entry	Additive (amount)	Solvent	t (°C)	Time (h)	Yield 2 (%)	Ref.
1	DMAP (1 equiv.)	CH_2_Cl_2_	r.t.	24	60	[13]
2	DMAP (1 equiv.)	THF	reflux	12	67	[13,14]
3	I_2_ (2 equiv.)	--	r.t.	12	37 ^a^	[15]
4	NaHCO_3_ (2 equiv.)	Dioxane	r.t.	12	traces ^a^	[16]
5	NaOH (2 equiv.)	Dioxane	0–r.t.	12	traces ^a^	[17]

Reaction conditions: Compound **1** (0.5 mmol), Boc)_2_O (1 mmol), solvent (5 mL), additive; ^a^ Determined by GC analysis; r.t. = room temperature.

**Scheme 3:**
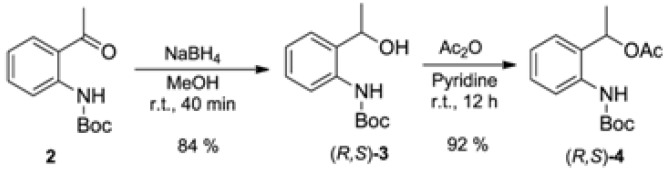
Synthesis of racemic compounds **3** and **4**.

The reduction of *tert*-butyl (2-acetylphenyl)carbamate (**2**) with NaBH_4_ gave (*R,S*)-*tert*-butyl 2-(1-hydroxyethyl)phenylcarbamate (**3**) in 84% yield (Scheme 3). The acylated derivative (*R,S*)-**4** was efficiently synthesized from (*R,S*)-**3** and acetic anhydride (92% yield, Scheme 3).

### 2.2. Enzymatic Kinetic Resolution of the (R,S)-tert-butyl 2-(1-Hydroxyethyl)*phenylcarbamate (**3**)*

#### 2.2.1. Screening of Lipases for Kinetic Resolution of (*R,S*)-**3**

A screening set with 12 different lipases was carried out, looking for a enzyme able to mediate the transesterification of (*R,S*)-**3** with high enantioselectivity and conversion in a short reaction time (Table 2).

**Table 2:** Screening of lipases for kinetic resolution of (*R,S*)-**3c**. 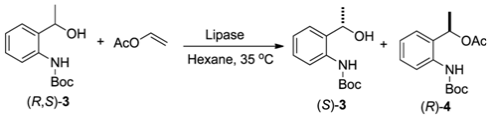

Entry	Lipase	Time (h)	c ^a^(%)	*ee *^b^ (%) (*S*)-3 (*R*)-4		*E * ^c^
1	*Candida Antarctica* (Novozym^®^ 435; immobilized on acrylic resin)	12	47	88	>99	>200
2		24	50	>99	>99	>200
3		48	51	>99	95	>200
4	*Pseudomonas cepacia* (immobilized on ceramics)	12	33	49	>99	>200
5		24	44	77	>99	>200
6		48	49	95	>99	>200
7	*Pseudomonas cepacia* (immobilized on diatomite)	12	16	19	>99	>200
8		24	26	34	>99	>200
9		48	36	56	>99	>200
10	*Candida rugosa*	12	14	13	81	10
11		24	17	17	81	11
12		48	21	21	80	11
13	*Candida cylindracea*	12	12	11	84	12
14		24	15	14	81	10
15		48	18	17	80	10
16	*Candida *sp. (Novozymes^®^ CALB L)	24	<5 ^d^	nd	nd	nd
17	*Thermomyces lanuginosus*	24	<5 ^d^	nd	nd	nd
18	*Rhizomucor miehei*	24	<5 ^d^	nd	nd	nd
19	Porcine pancreas lipase	24	<5 ^d^	nd	nd	nd
20	*Aspergillus niger*	24	<5 ^d^	nd	nd	nd
21	*Pseudomonas fluorescens*	24	<5 ^d^	nd	nd	nd
22	*Penicillium camemberti*	24	<5 ^d^	nd	nd	nd
23	*Mucor javanicus*	24	<5 ^d^	nd	nd	nd
24	*Pseudomonas cepacia*	24	<5 ^d^	nd	nd	nd

Reaction conditions: Compound (*R*,*S*)-**3** (0.25 mmol), lipase (20 mg), vinyl acetate (1 mmol), hexane (1 mL), 35 °C, 160 rpm; ^a^ conversion: c = 100 × (*ee_s_*/*ee_s_* + *ee_p_*); ^b^ enantiomeric excess: determined by HPLC analysis; ^c^ Enantiomeric ratio: *E* = ln{[*ee**_P_*** (1 − *ee**_S_***)]/(*ee**_P_*** + *ee**_S_***)}/ln{[*ee**_P_*** (1 + *ee**_S_***)]/(*ee**_P_*** + *ee**_S_***)}; ^d^ determined by GC analysis; nd: not determined due to low conversion.

Among the different type of lipases that were used as biocatalysts in the transesterification reaction, CAL-B presented high values of both conversion and enantioselectivity (Entries 1–3). After 12 h CAL-B-catalyzed reaction showed 47% conversion, high enantiomeric excess (*ee*) for (*S*)-**3** (88%) and (*R*)-**4** (>99%), and an enantiomeric ratio (*E*) higher than 200 (Entry 1). After 24 h, the conversion increased to 50% and both (*S*)-**3** and (*R*)-**4** were obtained with *ee* > 99% (Entry 2). After 48 h, the conversion was higher than 50% and consequently the *ee* of (*R*)-**4** dropped to 95%. Based on these results, 24 h was selected as the most appropriate time to interrupt the kinetic resolution process.

*Pseudomonas cepacia* lipases also presented interesting results, but slightly inferior to those of CAL-B. For *P. cepacia* immobilized on ceramics, we observed high *ee* for (*R*)-**4** (>99%) and *E* > 200 after 12 h (Entry 4). But, even after 48 h the conversion did not reach 50% and consequently the maximum *ee* for (*S*)-**3** was 95% (Entry 6). For *P. cepacia* immobilized on diatomite, the conversion was lower than 40% and 56% *ee* for (*S*)-**3** (Entry 9).

Lipases from *Candida rugosa*, *Candida cylindracea*, *Candida sp.*, *Thermomyces lanuginosus*, *Rhizomucor miehei*, porcine pancreas, *Aspergillus niger*, *Pseudomonas fluorescens*, *Penicillium camemberti*, *Mucor javanicus*, *Pseudomonas cepacia* showed discouraging results (Entries 10–24), including cases in which the conversion was lower than 5% (Entries 16–24). Then, the CAL-B was selected as the appropriate lipase to be applied to the next studies.

#### 2.2.2. Influence of Solvent in the Kinetic Resolution of (*R,S*)-**3**

The solvent influence on EKR of (*R,S*)-**3** was also investigated (Table 3). The results demonstrated that the reaction in hexane presented high conversion (50%) and excellent enantioselectivity. For those reactions in toluene (Entries 3 and 4) and methyl *tert*-butyl ether (Entries 5 and 6) slightly inferior results were observed, in comparison with hexane. On the other hand, THF, CHCl_3_, *i*-PrOH and *i*-BuOH (Entries 7–10) showed a dramatic influence on the conversion, which resulted in low values, <30%.

#### 2.2.3. Influence of Temperature in the Kinetic Resolution of (*R,S*)-**3**.

The temperature influence on EKR of (*R,S*)-**3** (Table 4) was also studied. Different reaction time was also evaluated (12, 16, 20 and 24 h). In this study, we found that at 25 °C the perfect kinetic resolution of (*R,S*)-**3** was achieved after 24 h (Entry 4). At 35 °C, the optimal values were achieved after 16 h (Entry 6). By increasing the temperature to 40 °C, the desired results were observed in 12 h (Entry 9). The same reaction-time tendency was verified at 50 °C. Therefore, 40 °C was chosen as the best temperature, since a reasonable reaction time with a relative low temperature can be used to obtain an excellent KR of (*R,S*)-**3**.

**Table 3:** Influence of solvent in the lipase-catalyzed transesterfication of (*R,S*)-**3**. 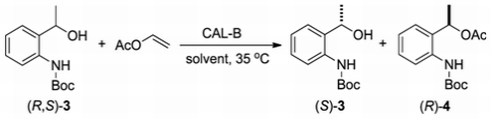

Entry	Solvent	Time (h)	c ^a^ (%)	*ee*^b^ (%) (*S*)-3 (*R*)-4		*E * ^c^
1	Hexane	12	47	88	>99	>200
2		24	50	99	>99	>200
3	Toluene	12	38	61	>99	>200
4		24	47	87	>99	>200
5	Methyl *tert*-butyl ether (MTE)	12	42	73	>99	>200
6		24	49	94	>99	>200
7	Tetrahydrofuran (THF)	24	<30 ^d^	nd	nd	nd
8	Cloroform (CHCl_3_)	24	<30 ^d^	nd	nd	nd
9	Isobutylic alcohol (*i*-BuOH)	24	<30 ^d^	nd	nd	nd
10	Diethylic ether (Et_2_O)	24	<30 ^d^	nd	nd	nd

Reaction conditions: Compound (*R*,*S*)-**3** (0.25 mmol), CAL-B (20 mg), vinyl acetate (1 mmol), solvent (1 mL), 35 °C, 160 rpm; ^a^ conversion: c = 100 × (*ee_s_*/*ee_s_* + *ee_p_*); ^b^ enantiomeric excess: determined by HPLC analysis; ^c^ Enantiomeric ratio: *E* = ln{[*ee**_P_*** (1 − *ee**_S_***)]/(*ee**_P_*** + *ee**_S_***)}/ln{[*ee**_P_*** (1 + *ee**_S_***)]/(*ee**_P_*** + *ee**_S_***)}; ^d^ determined by GC analysis; nd: not determined due to low conversion.

**Table 4:** Influence of temperature in the lipase-catalyzed transesterfication of (*R,S*)-**3**. 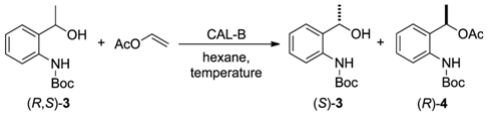

Entry	Temperature (°C)	Tempo (h)	c ^a^ (%)	*ee*^b^ (%) (*S*)-3 (*R*)-4		*E* ^c^
1	25	12	45	80	>99	>200
2	25	16	46	86	>99	>200
3	25	20	49	95	>99	>200
4	25	24	50	>99	>99	>200
5	35	12	48	89	>99	>200
6	35	16	50	>99	>99	>200
7	35	20	50	>99	>99	>200
8	35	24	50	>99	>99	>200
9	40	12	50	>99	>99	>200
10	40	16	50	>99	>99	>200
11	40	20	51	>99	98	>200
12	40	24	52	>99	97	>200
13	50	12	50	>99	98	>200
14	50	16	50	>99	98	>200
15	50	20	52	>99	97	>200
16	50	24	53	>99	94	>200

Reaction conditions: Compound (*R*,*S*)-**3** (0,25 mmol), CAL-B (20 mg), vinyl acetate (1 mmol), hexane (1 mL), 160 rpm; ^a^ conversion: c = 100 × (*ee_s_*/*ee_s_* + *ee_p_*); ^b ^enantiomeric excess: determined by HPLC analysis; ^c^ Enantiomeric ratio: *E* = ln{[*ee**_P_*** (1 − *ee**_S_***)]/(*ee**_P_*** + *ee**_S_***)}/ln{[*ee**_P_*** (1 + *ee**_S_***)]/(*ee**_P_*** + *ee**_S_***)}.

#### 2.2.4. Study of the Ratio of Enzyme to Substrate for Kinetic Resolution of (*R,S*)-**3**

The ratio enzyme/substrate was also investigated for EKR of (*R*,*S*)-**3** at 40 °C and 12 h (Table 5). It was observed that 10 mg of CAL-B was not enough to reach 50% of conversion (Entry 3). By using 20 and 40 mg the desired result was achieved just at the end of the experiments (Entries 6 and 9). However, the reaction with 80 mg of CAL-B showed 50% of conversion after 8 h (Entry 11) and for the reaction with 100 mg only 6 h were needed to obtain the desired results (Entry 13), but we considered the ratio of 100 mg CAL-B to 0.25 mmol substrate impracticable, so 20 mg was chosen as the optimal amount to achieve excellent values of conversion and enantiomeric excess.

**Table 5:** Study of ratio CAL-B/substrate for kinetic resolution of (*R,S*)-**3**. 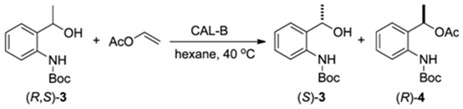

Entrada	Massa(mg)	Tempo (h)	c(%) ^a^	*ee* (%) ^b^ 3 4		*E * ^c^
1	10	6	30	63	>99	>200
2	10	8	39	75	>99	>200
3	10	12	45	90	>99	>200
4	20	6	40	84	>99	>200
5	20	8	45	93	>99	>200
6	20	12	50	>99	>99	>200
7	40	6	43	86	>99	>200
8	40	8	48	97	>99	>200
9	40	12	50	>99	>99	>200
10	80	6	48	97	>99	>200
11	80	8	50	>99	>99	>200
12	80	12	51	>99	97	>200
13	100	6	50	>99	>99	>200
14	100	8	50	>99	>99	>200
15	100	12	52	>99	95	>200

Reaction conditions: Compound (*R*,*S*)-**3** (0.25 mmol), CAL-B, vinyl acetate (1 mmol), hexane (1 mL), 40 °C, 160 rpm; ^a^ conversion: c = 100 × (*ee_s_*/*ee_s_* + *ee_p_*); ^b^ enantiomeric excess: determined by HPLC analysis; ^c^ Enantiomeric ratio: *E* = ln{[*ee**_P_*** (1 − *ee**_S_***)]/(*ee**_P_*** + *ee**_S_***)}/ln{[*ee**_P_*** (1 + *ee**_S_***)]/(*ee**_P_*** + *ee**_S_***)}.

**Scheme 4:**
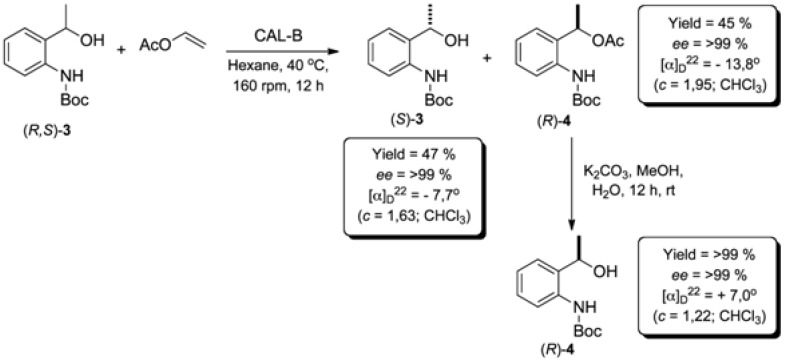
Synthesis of (*R*)- and (*S*)-**3**.

In order to obtain the compounds (*S*)-**3** and (*R*)-**3** and to assign the absolute configuration, a reaction on a preparative scale (5 mmol) was carried out. After quenching the reaction, the compounds (*S*)-**3** and (*R*)-**4** were separated by flash gel column chromatography. Then, the ester (*R*)-**4** was submitted to a hydrolysis reaction to give the alcohol (*R*)-**3** (Scheme 4). In this way, both enantiomers of **3** were obtained in high enantiomeric purity (*ee* > 99%) and yields (>45%).

The absolute configuration of the compound **3** was indirectly attributed after deprotection of the amino group of (−)-(*S*)-**3** [18]. Then, the optical rotation of the resulting amino-alcohol **5** was measured, and by comparison with literature data [18] its absolute configuration was attributed to (*S*)-**5** (Table 6). Consequently, the configuration of the *N*H-Boc protected precursor was also attributed to (*S*)-**3**.

**Table 6:** Assignment of the absolute configuration of *tert*-butyl 2-(1-hydroxyethyl)phenylcarbamate (**3**). 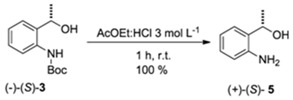

#	*ee* (%)	[a]_D_
Literature [18]	93	+52, 5 (*c* = 1,0; CHCl_3_)
This work	>99	+63,1 (*c* = 1,1; CHCl_3_)

## 3. Experimental Section

Commercially available materials were used without further purification. Lipase from *Candida antarctica* (fraction B, CAL-B) immobilized, and commercially available as Novozym® 435 was kindly donated by Novozymes Latin America Ltda. All solvents were HPLC or ACS grade. Solvents used for moisture sensitive operations were distilled from drying reagents under a nitrogen atmosphere: THF was distilled from Na/benzophenone.

Analytical thin-layer chromatography (TLC) was performed using aluminum-backed silica plates coated with a 0.25 mm thickness of silica gel 60 F_254_ (Merck), visualized with an ultraviolet light (l = 254 nm), followed by exposure to *p*-anisaldehyde solution or vanillin solution and heating. Standard chromatographic purification methods were followed using 35–70 mm (240–400 mesh) silica gel purchased from Acros Organics^®^.

Nuclear magnetic resonance (NMR) spectra were recorded on a Bruker AC 200 spectrometer at operating frequencies of 200 (^1^H-NMR) and 50 MHz (^13^C-NMR). The ^1^H-NMR chemical shifts are reported in ppm relative to TMS peak. The data are reported as follows: chemical shift (δ), multiplicity (s = singlet, d = doublet, t = triplet, qd = quadruplet, dd = double dublet, td = triple dublet, m = multiplet), and coupling constant (*J*) in Hertz and integrated intensity. The ^13^C-NMR chemical shifts are reported in ppm relative to CDCl_3_ signal.

Reaction products were analyzed by a Shimadzu model GC-17A (FID) gas chromatograph equipped with a J&W Scientific HP5 column (30 m × 0.25 mm I.D.; 0.25 µm). The chromatographic conditions were as follows: Oven temperature initiated at 50 °C and increased at 10 °C/min; run time 20 min; injector temperature 230 °C; detector temperature 250 °C; injector split ratio 1:20; hydrogen carrier gas at a pressure of 100 kPa. The enantiomeric excesses of the products were determined by HPLC analyses performed in a Shimadzu model SPD-10Av instrument with UV-Vis detector (deuterium lamp 190–600 nm) and equipped with a Chiralcel^®^ OD-H column (25 cm × 0.46 cm I.D.; Daicel Chemical Ind.) eluted with *n*-hexane (60%) and 2-propanol (99:1).

High-resolution mass spectra (HRMS) were acquired using a Bruker Daltonics MicroTOF instrument, operating in the electrospray ionization (ESI) mode.

Infrared spectra were recorded from KBr discs or from a thin film between NaCl plates on FTIR spectrometer (Bomem Michelson model 101). Absorption maxima (ν_max_) are reported in wavenumbers (cm^−1^).

Optical rotations were measured on a Perkin Elmer-343 digital polarimeter in a 1 mL cuvette with a 1 dm pathlength. All values are reported in the following format: [α]_D_(temperature of measurement) = specific rotation (concentration of the solution reported in units of 10 mg sample per 1 mL solvent used).

### 3.1. Synthesis of tert-butyl (2-Acetylphenyl)carbamate *(2)* (Adapted from References [13,14])

To a solution of the 1-(2-aminophenyl)ethanone (**1**, 1.35 g, 10 mmol) in anhydrous THF (100 mL) Boc)_2_O (6.48 g, 30 mmol) was added, followed by DMAP (122 mg, 1 mmol). The solution was stirred under reflux for 12 h then concentrated to dryness and partitioned between 0.5 mol L^−1^ HCl (100 mL) and EtOAc (100 mL). The aqueous layer was extracted with EtOAc (2 × 100 mL) and the combined organic phases were washed with brine (50 mL), dried over MgSO_4_, filtered and concentrated to afford the crude *tert*-butyl (2-acetylphenyl)carbamate (**2**) and the di-Boc derivative products. These compounds were separated by flash silica gel column chromatography eluted with hexane/EtOAc 9:1. (2-Acetylphenyl)carbamate (**2**) and the di-Boc derivative were isolated in 45% and 31% yields, respectively. The di-Boc compound (1.00 g) was dissolved in CH_2_Cl_2_ (100 mL) and Amberlyst 15 resin (1.00 g) was added. The mixture was stirred for 24 h in an orbital shaker. Then, the solvent was removed and the residue filtered through a silica gel column with hexane/EtOAc 9:1. The compound **2** was obtained in 67% yield. ^1^H-NMR (200 MHz, CDCl_3_); d (ppm): 10.95 (s, 1H); 8.46 (d, 1H, *J* = 8.3 Hz); 7.84 (dd, 1H, *J*_A_ = 7.9 Hz; *J*_B_ = 1.32 Hz); 7.50 (td, 1H, *J*_A_ = 8.3 Hz; *J*_B_ = 1.3 Hz); 7.01 (t, 1H, *J* = 7.9 Hz); 2.64 (s, 3H); 1.53 (s, 9H). ^13^C-NMR (50 MHz, CDCl_3_); d (ppm): 202.5; 153.4; 142.0; 135.2; 131.9; 121.6; 121.2; 119.4; 80.7; 28.8. IV (KBr), cm^−1^: 3432; 2947; 1713; 1656; 1562; 1239; 1108; 739. HRMS (ESI), [M+Na]^+^: Calculated for C_13_H_17_NO_3_Na: 258.1106. Found: 258, 1104.

### 3.2. Synthesis of Racemic tert-butyl (2-(1-Hydroxyethyl)phenyl)carbamate [(*R,S*)-**3**]

To a solution of *tert*-butyl (2-acetylphenyl)carbamate (**2**, 1.175 g, 5 mmol) in methanol (50 mL) NaBH_4_ (0.21 g, 5.5 mmol) at 0 °C was added. After adding NaBH_4_, the ice bath was removed and the solution was stirred at room temperature for 2 h then concentrated to dryness. To residue water (30 mL) was added and the pH adjusted to 6.0. In the sequence the mixture was extracted with CH_2_Cl_2_ (3 × 15 mL), dried over MgSO_4_, filtered and concentrated to afford the crude *tert*-butyl (2-(1-hydroxyethyl)phenyl)carbamate (**3**). This was purified by flash silica gel column chromatography eluted with hexane/EtOAc 9:1 to afford **3** in 84% yield. ^1^H-NMR (200 MHz, CDCl_3_);d (ppm): 8.01 (s, 1H); 7.90 (d, 1H, *J* = 8.3 Hz); 7.26 (t, 1H, *J* = 7.5 Hz); 7.14 (d, 1H, *J* = 7.5 Hz); 7,00 (t, 1H, *J* = 7.5 Hz); 4,95 (qd, 1H, *J* = 6.6 Hz); 1.54 (m, 12H). ^13^C-NMR (50 MHz, CDCl_3_); d (ppm): 153.5; 136.7; 132.7; 127.9; 126.4; 122.9; 121.4; 80.0; 69.5; 28.2; 22.1. IR (film), cm^−1^: 3457; 3343; 2979; 1761; 1727; 1524; 1449; 1254. HRMS (ESI), [M+Na]^+^: Calculated for C_13_H_19_NO_3_Na: 260.1263. Found: 260.1262.

### 3.3. Synthesis of Racemic 1-(2-((tert-Butoxycarbonyl)amino)phenyl)ethyl acetate [(*R,S*)-**4**]

To a solution of the (2-(1-hydroxyethyl)phenyl)carbamate (**3**, 237 g, 1 mmol) in pyridine (2 mL) was added Ac_2_O (0.10 g, 1 mmol). The solution was stirred at room temperature overnight then diluted in EtOAc (20 mL) and washed with CuSO_4_ (5 mL portions) to the complete removal of the pyridine. The organic phase was dried over MgSO_4_, filtered and concentrated to afford the crude 1-(2-((*tert*-butoxycarbonyl)amino)phenyl)ethyl acetate (**4**). The crude material was purified by flash silica gel column chromatography eluted with hexane/EtOAc 9:1 to afford **4** in 92% yield. ^1^H-NMR (200 MHz, CDCl_3_); d (ppm): 7.78 (d, 2H, *J* = 8.1 Hz); 7.66 (s, 1H); 7.32 (m, 2H); 7.12 (td, 1H, *J*_A_ = 7.5 Hz; *J*_B_ = 0.8 Hz); 5.98 (qd, 1H, *J* = 6.4 Hz); 2.0 (s, 3H); 1.61 (d, 3H, *J* = 6.4 Hz); 1.5 (s, 9H). ^13^C-NMR (50 MHz, CDCl_3_); d (ppm): 170.2; 152.8; 135.1; 130.7; 128.1; 126.3; 123.6; 122.9; 79.3; 68.1; 27.6; 20.3; 20.0. IR (film), cm^−1^: 3432; 3338; 2980; 1731; 1591; 1519; 1453; 1241; 1160. HRMS (*ESI*), [M+Na]^+^: Calculated for C_15_H_21_NO_4_Na: 302.1368. Found 302.1364.

### 3.4. Enzymatic Kinetic Resolution of the (R,S)-tert-butyl 2-(1-Hydroxyethyl)phenylcarbamate [(*R,S*)-**3**]

To solution of racemic *tert*-butyl (2-(1-hydroxyethyl)phenyl)carbamate (**3**, 1.185 g; 5 mmol) in hexane (20 mL), CAL-B (Novozym^®^ 435; 400 mg) and vinyl acetate (1.72 g; 20 mmol) were added. The mixture was stirred in an orbital shaker at 40 °C for 12 h (160 rpm). Following that, the enzyme was filtered off and washed with dichloromethane (3 × 20 mL). The solvent was removed under reduced pressure and the residue was purified by flash silica gel column chromatography eluted with hexane/EtOAc 9:1 to afford (*S*)-**3** (*ee* > 99%) in 47% yield and (*R*)-**4** (*ee* > 99%) in 45% yield.

### 3.5. HPLC Analysis of (S)- and (R)-tert-butyl (2-(1-Hydroxyethyl)phenyl)carbamate (**3**)

HPLC conditions: Chiralcel^®^ OD-H column, *n*-hexane/*i*-PrOH (99:1), 1.0 mL min^−1^, 254 nm UV detector. (*S*)-**3**: isolated yield = 45%; retention time: 23.7 min; *ee* > 99%; [α]_D_^22^ = −7.7 (*c *= 1.63; CHCl_3_). (*R*)-**3**: Isolated yield = 45%; retention time: 29.2 min; *ee* > 99%; [α]_D_^22^ = 7.0 (*c *= 1.22; CHCl_3_).

### 3.6. General Procedure to Remove Boc-Protecting Group (Adapted from Reference [19])

To a mixture of AcOEt:HCl 3 mol L^−1^ 1:1 (5 mL), *N*-Boc protected compound (1 mmol) was added. The mixture was stirred at room temperature for 1 h. After that, the solvent was removed under vacuum. The residue was dissolved in CH_2_Cl_2_ (10 mL) and washed with saturated NaHCO_3_ solution (3 × 3 mL). Then, the organic layer was dried over MgSO_4_, filtered and concentrated to dryness under vacuum. The product was obtained in quantitative yield without further purification.

## 4. Conclusions

In summary, we have described an efficient methodology to obtain (*R*)- and (*S*)-*tert*-butyl 2-(1-hydroxyethyl)phenylcarbamates in enantiopure form (*ee* > 99%), using a kinetic resolution process mediated by lipase as a biocatalyst. Both enantiomers can be employed in the preparation of organochalcogenanes for further application in biological studies.
